# Sustainable isomaltulose production in *Corynebacterium glutamicum* by engineering the thermostability of sucrose isomerase coupled with one-step simplified cell immobilization

**DOI:** 10.3389/fmicb.2022.979079

**Published:** 2022-08-10

**Authors:** Mengkai Hu, Fei Liu, Zhi Wang, Minglong Shao, Meijuan Xu, Taowei Yang, Rongzhen Zhang, Xian Zhang, Zhiming Rao

**Affiliations:** Key Laboratory of Industrial Biotechnology, Ministry of Education, School of Biotechnology, Jiangnan University, Wuxi, China

**Keywords:** thermostability, isomaltulose, sucrose isomerase, one-step simplified immobilization, repeated batches

## Abstract

Sucrose isomerase (SI), catalyzing sucrose to isomaltulose, has been widely used in isomaltulose production, but its poor thermostability is still resisted in sustainable batches production. Here, protein engineering and one-step immobilized cell strategy were simultaneously coupled to maintain steady state for long-term operational stabilities. First, rational design of *Pantoea dispersa* SI (PdSI) for improving its thermostability by predicting and substituting the unstable amino acid residues was investigated using computational analysis. After screening mutagenesis library, two single mutants (PdSIV280L and PdSIS499F) displayed favorable characteristics on thermostability, and further study found that the double mutant PdSIV280L/S499F could stabilize PdSIWT better. Compared with PdSIWT, PdSIV280L/S499F displayed a 3.2°C-higher *T*_*m*_, and showed a ninefold prolonged half-life at 45°C. Subsequently, a one-step simplified immobilization method was developed for encapsulation of PdSIV280L/S499F in food-grade *Corynebacterium glutamicum* cells to further enhance the recyclability of isomaltulose production. Recombinant cells expressing combinatorial mutant (RCSI2) were successfully immobilized in 2.5% sodium alginate without prior permeabilization. The immobilized RCSI2 showed that the maximum yield of isomaltulose by batch conversion reached to 453.0 g/L isomaltulose with a productivity of 41.2 g/l/h from 500.0 g/L sucrose solution, and the conversion rate remained 83.2% after 26 repeated batches.

## Introduction

Currently, excessive sugar intake has led to people’s weight gain and its associated health problems such as hyperlipidemia, hypertension, and diabetes (Olszewski et al., 2019). Exploring low-calorie functional sweeteners for food ingredients and additives has become a research hotspot (Novotny et al., 2019; Van Laar et al., 2021). Isomaltulose, an isomer of sucrose, is a reducing disaccharide. It exists in natural molasses in a small amount and has 45 percent of the sweetness of sucrose, but is non-toxic and non-cariogenic (Barea-Alvarez et al., 2014). Therefore, isomaltulose is an ideal sucrose substitute and is a Food Drug Administration certified healthy sugar, and its addition and consumption are not restricted (Linaa et al., 2002). In addition, isomaltulose has many beneficial healthcare functions and physiological properties, including inhibiting elevated blood sugar levels (Maresch et al., 2017), inhibiting fat accumulation (Lee et al., 2020), improving anti-fatigue ability (Stevenson et al., 2017), and maintaining the intestinal micro ecological balance (Kendall et al., 2018). However, the process of chemically synthesizing isomaltulose produces by-products and chemical waste, increasing the cost of product separation and wastewater treatment (Zhang et al., 2017). Therefore, the preparation of isomaltulose by biotransformation technology has been widely investigated in recent years (Liu et al., 2020).

Sucrose isomerase (SI, EC 5.4.99.11), be known as isomaltulose synthase, converts sucrose into isomaltulose or trehalulose along with glucose and fructose (Mu et al., 2014). Current investigations of SI are mainly in the mining of novel genes and property characterizations of SIs. Those currently-reported SIs showed limited thermostability during the biocatalysis process, such as the SI of *Klebsiella* sp. LX3, which has a 1.8 min half-life at 50°C (Li et al., 2011), SI of *Klebsiella pneumonia* lost its 40% relative activity after incubating at 50°C for 20 min (Aroonnual et al., 2007), and SI of Erwinia sp was completely inactivated after 24 h incubation at 30°C (Kawaguti and Harumi Sato, 2010). Based on our comparative analysis, PdSI from *Pantoea dispersa* have strong ability to converting sucrose to isomaltulose, and the reaction conditions are more suitable for industrial production than others sources, but its thermostability is still unsatisfactory in the industrial applications (Li et al., 2017; Zhang et al., 2019; Zheng et al., 2019). Therefore, modification at the molecular level to improve the thermostability of PdSI should be further investigated.

Protein engineering has been shown to be an effective approach to enhance the thermostability of enzymes and is subdivided into directed evolution, semi-rational design, and rational design (Stepankova et al., 2013). Although the irrational and semi-rational designs were powerful in enzyme modification at elevated temperatures (Tizei et al., 2016; Arnold, 2019), they are time-consuming and laborious. In contrast, rational design based on computer-aided has greatly accelerated scientific research’s speed and success rate (Cui et al., 2020). Fold X, one of the most reliable computational design predictors, has been developed to predict beneficial substitutions related to thermal stability by rapidly evaluating the Gibbs free energy difference (ΔΔG) (Guerois et al., 2002; Schymkowitz et al., 2005). Recently, FoldX has been used to improve the thermostability of many enzymes. Luo et al. (2016) obtained the best variant PoOPH_*M9*_ with thermostability (T_50_^15^) of 67.6°C via hierarchical iteration mutagenesis. Bi et al. (2020) engineered thermophilic pullulanase using FoldX predictor, the *T*_*m*_ of mutant G692M increased by 3.8°C, and the half-life is 2.1-fold longer than the wild-type at 70°C. Wang et al. (2020) constructed a mutant (S142A/D217V/Q239F/S250Y) based on the FoldX algorithm, and the half-life of the combination mutant increased 41.7-fold at 60°C. Thus, *in silico* energy calculations (FoldX) may provide a clear guide for the molecular engineering of SI.

Besides, the production method of isomaltulose also affects the thermostability of SI to a certain extent. Immobilized enzymes or cells have become the main biocatalytic route for isomaltulose production, but immobilized enzymes still face high cost and tedious operation steps such as cell disruption and protein purification (Wu et al., 2015; Zhang et al., 2019). In contrast, immobilized cells have become an alternative approach. However, endotoxin or toxic cell wall pyrogens of non-food-grade hosts would be an obstacle to the green synthesis of isomaltulose. To solve the potential safety hazards, some researchers have introduced SIase genes into non-pathogenic hosts, including *Lactococcus lactis* MG1363 (Park et al., 2010), *Bacillus subtilis* WB800 (Wu et al., 2017), *Saccharomyces cerevisiae* (Lee et al., 2011), and *Yarrowia lipolytica* S47 (Zhang et al., 2018). However, *L. lactis* MG1363 (Park et al., 2010) exhibited a low expression level of SI (100 μg/mL), and *S. cerevisiae* (Lee et al., 2011) and *Y. lipolytica* (Zhang et al., 2018) grew slowly (48–96 h). *Corynebacterium glutamicum* ATCC13032 is listed as a “generally recognized as safe” microorganism and has been successfully used as a host for producing food compounds efficiently, like amino acids, vitamins, organic acids, and rare sugars (Shin et al., 2016). As far as we know, *C. glutamicum* has currently been used as a food-grade expression platform to produce the rare sugars and sugar alcohols, such as D-Tagatose (Shin et al., 2016), D-allulose (Yang et al., 2019), D-mannitol (Baumchen and Bringer-Meyer, 2007) and so on. However, due to the rigid cell wall structure of Gram-positive bacteria such as *C. glutamicum* and *Lactobacillus*, the catalytic efficiency is often affected by the transmembrane transport of substrates or products. Traditionally, cells need to be permeabilized before immobilization (Shin et al., 2016; Bober and Nair, 2019), but this process can cause cell lysis and waste time to prepare immobilized cells. Therefore, development of a one-step simplified immobilized cell method has also become particularly important.

Herein, in order to obtain robust SI from a small mutation library via rational design, computational design software (FoldX5) combined with conservation analysis and functional region assessment was employed to predict potential candidates. Then, Differential Scanning Fluorimetry (DSF) and molecular dynamic simulation (MD) were used to evaluate the changes in the thermostability and elucidate the mechanism, respectively. Finally, the best variant was intracellularly overexpressed in the food-grade strain *C. glutamicum*, and recombinant cells were further immobilized by one-step simplified immobilization method for the sustainable production of isomaltulose. Taken together, combined strategies involving computational-aided design, rational engineering and immobilized cells in this work provide a strategy to improve its performance in industrial applications. As far as we know, this is the first report of the maximum batches of isomaltulose production using an immobilized engineered food-grade host.

## Materials and methods

### Reagents and enzymes

Sucrose, isomaltulose, and high-performance liquid chromatography (HPLC)-grade acetonitrile were bought from Aladdin (Shanghai, China). PrimSTAR Max DNA Polymerase, restriction enzymes, protein markers, and *Dpn*I were purchased from TaKaRa (Dalian, China). Isopropyl-β-D-thiogalactopyranoside and chloramphenicol were supplied by Yuanye Bio-Technology (Shanghai, China). Plasmid Mini Preparation Kit was provided by Beyotime (Shanghai, China). Other reagents were purchased from Sinopharm Chemical Reagent (Shanghai, China) unless otherwise noted.

### Plasmids, strains, and medium

All plasmids and strains used in this study could be found in Supplementary Table 1. The original sequence of SI gene from the *Pantoea dispersa* UQ68J (*Pdsi*) (GenBank accession number: AY223549) without signal peptide was synthesized and sequenced by Suzhou GENEWIZ Company, and the C-terminal’s 6xHis tag of *Pdsi* was used for protein purification. The *Pdsi* gene was incorporated between *Hind*III and *EcoR*I sites of the *Escherichia coli/C. glutamicum* shuttle plasmid pXMJ19 to generate pXMJ19-*pdsi*. *E. coli* strain JM109 was used as a gene cloning host to construct recombinant plasmids. *C. glutamicum* was used as an expression host to characterize enzymatic properties, and whole cells were used as biocatalysts for cell immobilization. *E. coli* cells were grown in LB medium at 37°C. *C. glutamicum* cells were grown on BHI medium at 30°C. Chloramphenicol was added if necessary to the final concentration of 25 μg/mL.

### Computational prediction for sucrose isomerase thermostability

As a starting point, the structure of wild-type (PdSIWT) and other mutants were modeled using the ERSI from *Erwinia rhapontici* NX5 (PDB: 4hph.1.A) as a template with 74.10% sequence identity by SWISS-MODEL (Xu et al., 2013), MolProbity (Chen et al., 2010) and PROCHECK (Laskowski et al., 1993) were applied for model evaluation, and evaluation results of PdSIWT are presented as Ramachandran plot (Supplementary Figure 1). Candidates for site-directed mutagenesis were identified based on ΔΔG changes. FoldX 3.0 algorithm was utilized to estimate the folding free energy of PdSIWT. A standardized script written in python was performed to change all positions of the protein sequence to other 19 amino acids. The relative folding free energy changes (ΔΔG = ΔG_*Mut*_ − ΔG_*WT*_) was calculated after each residue was mutated into the other amino acids.

### Site-directed mutagenesis PCR

The plasmid pXMJ19-*pdsi* was used as an amplification template to construct the SI mutants by using overlap extension PCR. The primers used could be found in Supplementary Table 2. Final amplification fragments were digested by the endonuclease *Dpn* I at 37°C for 1.5 h. Then the PCR mixture was chemically transformed into *E. coli* JM109. The sequenced plasmids were transformed into *C. glutamicum* cells by electroporation for protein expression.

### Expression and purification

The recombinant *C. glutamicum* strains were first cultivated into 10 mL BHI liquid medium supplemented with 25 μg/mL chloramphenicol at 30°C for overnight, and then 2% inoculation volume were transferred to 100 mL BHI medium. When the optical density at 600 nm was approximately 1.5, the expression of SI was induced with 0.5 mM IPTG at 30°C for another 20 h.

The cells were centrifuged (8,000 × *g*, 4°C) for 5 min, washed twice, and then resuspended in 10 mL of 50 mM citric acid-Na_2_HPO_4_ buffer (pH 6.0). Cells in suspension were sonicated for 20 min and centrifuged to remove cell debris. Subsequently, the soluble supernatant fractions were loaded onto a 1 mL Ni affinity column (GE Healthcare, HisTrap HP) after filtration, and the column was pre-equilibrated with 50 mM wash buffer (20 mM Tris and 500 mM NaCl, pH 7.4) before loading samples. Finally, the target protein was eluted using an elution buffer (20 mM Tris, 500 mM NaCl, and 300 mM imidazole, pH 7.4) with a linear gradient. Sodium dodecyl sulfate-polyacrylamide gel electrophoresis (SDS-PAGE) was used to analyze purified protein. The concentration of protein was determined through the Bradford method.

### Determination of enzyme activity

The isomaltulose-forming activity of PdSIWT and mutants was measured using 525 mM sucrose as substrate (pH 6.0). Specifically, 100 μL purified enzyme was incubated with 900 μL sucrose (584 mM) in 50 mM citric acid-Na_2_HPO_4_ buffer (pH 6.0), the reaction mixture was performed at 30°C for 10 min and was stopped by boiling at 100°C for 5 min. One unit (U) of SI activity was defined as the amount of SI required to catalyze the formation of 1 μmol isomaltulose per 1 min under the above conditions.

### Determination of optimal pH and temperature

The optimal pH value for enzyme activity was assayed in 50 mM citric acid- Na_2_HPO_4_ buffer (pH 4.0–8.0) at 30°C. The optimal temperature was determined between 20 and 50°C in buffer (pH 6.0). Purified enzyme of PdSIWT and mutants were incubated at 45°C to determine thermostability. Samples were taken at 20 min intervals and the residual activity was then determined. The original activity before incubating at 45°C was taken as 100%. Each assay was repeated three times.

### Determination of kinetic parameters

Kinetic parameters of purified enzymes were determined under standard assay conditions with sucrose as a substrate. The sucrose substrate concentrations were as follows: 14.6, 29.2, 58.4, 102.0, 146.0, 234.0, 292.0, and 584.0 mM, respectively. Then, *V*_*max*_ and *K*_*m*_ values were determined through regression fitting of experimental data using GraphPad Prism 8.0.

### Differential scanning fluorescence assay

The DSF procedure used in this research was slightly modified (Bi et al., 2020). *T*_*m*_ values were determined by monitoring the maximum relative fluorescence intensity after incubating the purified protein and SYPRO Orange dye together in PCR tubes at elevated temperatures in the real-time PCR machine. *T*_*m*_ values were measured at 1°C/min rate over the range of 25 ∼ 95°C. Three parallel samples were determined.

### Bioinformatics analysis

Molecular dynamic simulations (MD) were performed by using YASARA software^1^. Specifically, PdSIWT and mutant’s thermal fluctuations were analyzed using an Amber03 force field, and SI was surrounded by H_2_O containing 0.29% NaCl with pH 6.0 in a dodecahedron box. ConSurf Server was employed to identify proteins’ functional Regions (Berezin et al., 2004). Residue Interaction Network Generator was used to recognize various kinds of interactions introduced by the mutation in this study (Piovesan et al., 2016). ESPript 3.0 was mainly employed to analyze alignment sequence (Robert and Gouet, 2014).

### Batch culture

RCSI was cultivated into 200 mL of seed medium in a 1 L shake flask and grown at 30°C for 15 h with shaking at 220 rpm. The seed medium (g/L) consisted of 0.4 MgSO_4_⋅7H_2_O, 0.5 KH_2_PO_4_, 1.0 urea, 1.5 K_2_HPO_4_, 10.0 (NH_4_)_2_SO_4_, 50.0 corn syrup, 10.0 angel yeast, 40.0 glucose, and the initial pH of the seed medium was 7.0. The seed culture (200 mL) was inoculated into 1.8 L sterilized fermentation medium (g/L) [150.0 glucose, 5.0 corn syrup, 20.0 angel yeast, 30.0 (NH_4_)_2_SO_4_, 2.0 KH_2_PO_4_, 1.0 urea, 1.0 KCl, 0.5 MgSO_4_⋅7H_2_O, 0.02 ZnSO_4_⋅7H_2_O, 0.02 FeSO_4_⋅7H_2_O, and 0.02 MnSO_4_⋅H_2_O] of a 5 L fermenter (DIBIER, Shanghai, China). The temperature and pH were maintained at 30°C, 7.0, respectively. The final concentration of 0.5 mM IPTG was added to the fermenter to induce PDSI when the optical density reached 20 at 600 nm. The DO was kept at 30% (v/v) by coupling with the agitation speed. The 50% (V/V) ammonia solution was used to adjust the pH of the medium.

### Preparation of immobilized *C. glutamicum* cells by a one-step simplified immobilization method

For the immobilization of the recombinant *C. glutamicum* cells (RCSI), approximately 150 g cells (wet weight) were mixed in 1 L of 2.5% sodium alginate solution containing 0.1% Triton X-100. After thoroughly mixing, the mixture was slowly dropped into 8% (w/v) CaCl_2_ solution containing 25% sucrose to form 3.75 mm immobilized pellets using a needle. The pellets are stored at 4°C overnight in this solution, and then were washed with sterile distilled water to remove CaCl_2_. Immobilized *C. glutamicum cells* were then used for the production of isomaltulose. In addition, parameters related to cell immobilization, such as the concentration of sodium alginate and CaCl_2_ were optimized, mechanical strength was used to verify the hardness of immobilized pellets. Mechanical strength (g/cm^2^) was measured by pressing 20 immobilized particles on an electronic balance and reading the maximum pressure they can withstand when they were broken.

### Biosynthesis of isomaltulose using immobilized *C. glutamicum* cells in a 5 L fermenter

For biotransformation, immobilized *C. glutamicum* cells pellets (130 g/L) were washed three times with 50 mM citric acid-Na_2_HPO_4_ buffer (pH 6.0). The *C. glutamicum* cells pellets were then transferred into the 1.5 L reaction solution containing 500 g/L sucrose (pH 6.0, citric acid-Na_2_HPO_4_ buffer). The whole reaction process was carried out at 35°C for 11 h, and the speed is adjusted to 40 rpm to stir the reaction liquid in the catalytic process. Samples were collected at different time and further tested by HPLC. After each biotransformation, immobilized *C. glutamicum* cells pellets were recycled with simple centrifugation for the next batch.

### High-performance liquid chromatography analysis

Samples were determined by HPLC (Agilent 1260, United States) system equipped with a refractive index detector (RID) and separated by an NH_2_ column (DIKMA, platisil 5 μm NH_2_, 250 mm × 4.6 mm). The mobile phase was 80% acetonitrile at a flow rate of 1.0 mL⋅min^–1^ at 30°C, and RID temperature was controlled at 35°C. The amounts of sugar concentration were calculated via peak areas.

## Results and discussion

### Selection of the mutagenesis sites for improving thermostability of sucrose isomerase

**FIGURE 1:**
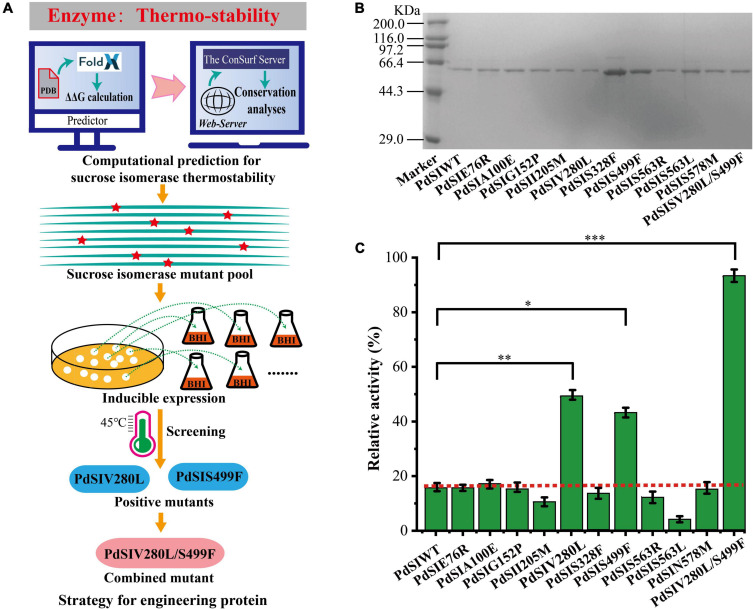
**(A)** The Flowchart for engineering sucrose isomerase. **(B)** SDS-PAGE shows the expression of mutants. **(C)** The residual activity of PdSIWT and its mutants after incubation at 45°C for 20 min. *p*-values are represented as follow: *P < 0.05, ^**^P < 0.01, ^***^P < 0.001.

To rationally design the most promising mutants, FoldX was used to screen variant PdSI by calculating the relative folding free energy changes (ΔΔG). When ΔΔG > 0, it means that the structure of the mutant is more unstable than that of the wild type. While ΔΔG < 0, the structure of the mutant is more stable. After calculation, ΔΔG values of all 10,982 single point mutations were obtained, and the mutation sites with ΔΔG < 0 were selected as the candidates. To further improve the prediction accuracy by the FoldX algorithm, an additional conservation analysis was performed to avoid point mutation of amino acids at conserved positions resulting in loss of enzyme activity (Li et al., 2018). The Consurf Server, as an essential tool for evolutionary conservation analyses, can automatically estimate the conserved degree of amino acids in homologous sequences of SI (Berezin et al., 2004). When the amino acid is marked with the letter “f” or “s,” it means that the amino acid is highly conserved in critical functional domains, that is, the residue is not suitable as a candidate for thermal stability modification. When the amino acid is marked with the letter “e” or “b,” the amino acid is variable in functional domains and then the residue can be considered as a candidate for thermal stability modification (Supplementary Figure 2). Finally, ten mutations were selected for the subsequent experimental study after computation-aided engineering: PdSIE76R, PdSIA100E, PdSIG152P, PdSII205M, PdSIV280L, PdSIS328F, PdSIS499F, PdSIS563R, PdSIS563L, and PdSIN578M (Table 1). The strategy of engineering SI thermo-stability is shown in Figure 1A.

**TABLE 1:** The ΔΔG values of candidate mutants computed by FoldX, and special activity of mutants.

Position	Original amino acid	Mutant amino acid	ΔΔG value (Kcal⋅mol^–1^)	Specific activity (U mg^–1^)
76	E	R	−1.94251	613 ± 3.3
100	A	E	−1.17737	620 ± 4.2
152	G	P	−2.26873	606 ± 3.7
205	I	M	−1.43737	438 ± 3.9
280	V	L	−1.68313	616 ± 3.5
328	S	F	−2.41961	533 ± 4.1
499	S	F	−1.57516	618 ± 2.7
563	S	L	−1.53936	282 ± 4.2
563	S	R	−1.57516	501 ± 4.2
578	N	M	−1.41972	595 ± 3.7

Each single point mutation was individually expressed in *C. glutamicum* to test whether these substitution mutations improved the thermostability of PdSIWT (Figure 1B). To obtain positive mutants quickly, the thermostability of mutants was evaluated by determining the residual activity after heat treatment at 45°C for 20 min. As shown in Figure 1C, PdSIWT retained 15.7% (98.4 U/mg) of its initial activity, whereas two positive mutants PdSIV280L and PdSIS499F retained 49.1% (302.5 U/mg) and 43.2% (267.0 U/mg) of their original activity (Table 1), respectively. However, thermostability of other mutants did not change or decrease significantly. These results demonstrated that PdSIV280L and PdSIS499F show better thermostability than PdSIWT. To assess the possible interaction between these two single points, double mutant PdSIV280L/S499F was constructed and investigated. PdSIV280L/S499F retained 93.3% (581.3 U/mg) of its initial activity after incubating at 45°C for 20 min.

### Enzymatic properties and kinetic analysis of PdSIV280L, PdSIS499F, and PdSIV280L/S499F

**FIGURE 2:**
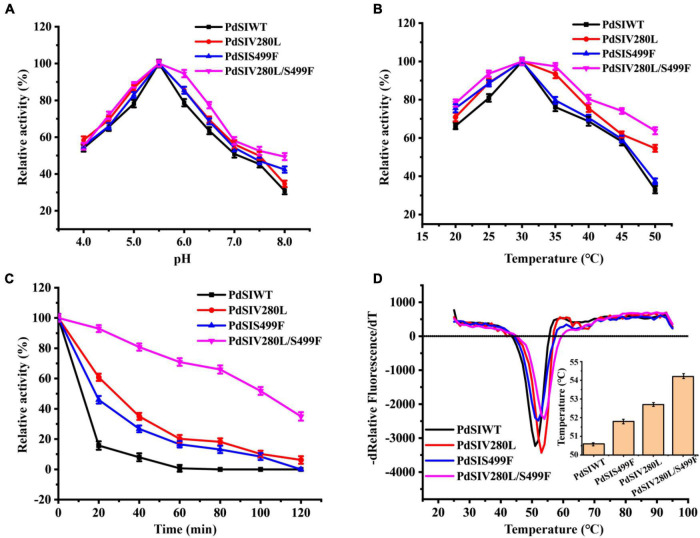
Effects of pH and temperature on the enzyme activity of PdSIWT and mutants. **(A)** The optimal reaction pH. **(B)** The optimal reaction temperature. **(C)** The thermostability of PdSIWT and positive mutants PdSIV280L, PdSIS499F, and PdSIV280L/S499F at 45°C. **(D)** The *T*_*m*_ of PdSIWT and mutants measured by DSF.

The thermostabilities and catalytic properties of the PdSIWT and positive mutants PdSIV280L, PdSIS499F, and PdSIV280L/S499F were further characterized. The optimal pH value of the three mutants was 5.5, similar to that of the PdSIWT (Figure 2A). Consistent with PdSIWT, these three positive mutants’ optimal temperature was 30°C, while they exhibited higher relative activity at the same temperature (Figure 2B). At 45°C, PdSIWT retained 58.1% of its maximum activity, whereas mutants PdSIV280L, PdSIS499F, and PdSIV280L/S499F retained 61.8, 59.4, and 74.1% of its maximum activities, respectively.

Then, changes in thermostability of these three mutants and PdSIWT were assessed by determining residual activities after different incubation times at 45°C. As shown in Figure 2C, the thermostability of mutants was significant than that of PdSIWT, and PdSIV280L/S499F displayed the most significant improvement. At 45°C, the t_1/2_ of PdSIWT was only 11.2 min. In contrast, the t_1/2_ of PdSIV280L, PdSIS499F, and PdSIV280L/S499F were 25.4, 21.5, and 100.0 min, 2.3, 1.9, and 8.9 times better than PdSIWT. These findings thus indicated that two amino acid substitutions (V280L and S499F) were beneficial to improve the thermostability of PdSIWT.

To further evaluate the thermodynamic stability of PdSIWT and its variants, the melting temperature (*T*_*m*_) was measured by DSF. As shown in Figure 2D, the *T*_*m*_ of PdSIWT was 50.6°C, while the *T*_*m*_ values of PdSIV280L, PdSIS499F, and PdSIV280L/S499F mutants were 52.7, 51.8, and 54.2°C, respectively. These results are consistent with the thermostability studies of the three positive mutants.

Kinetic parameters of the PdSIWT and its mutants were measured using different concentrations of sucrose as substrate. As listed in Table 2, three mutants showed slight differences in catalytic activity with PdSIWT. *K*_*m*_ and *k*_*cat*_/*K*_*m*_ of these mutants also changed slightly, indicating that point mutations have little influence on enzyme properties while improving the thermostability.

**TABLE 2:** Comparison of wild-type (WT) PDSI and mutants enzymatic properties.

Enzyme	*K*_*m*_ (mM)	*K*_*cat*_ (S^–1^)	*K*_*cat*_/*K*_*m*_ (S^–1^ mM^–1^)	*T*_*m*_ (°C)	*t*_1/2_(min) (45°C)	Special activity (U mg^–1^)
WT PDSI	42.1 ± 1.8	712 ± 6.1	16.7 ± 1.2	50.6 ± 0.1	11.2	627 ± 2.1
V280L	44.1 ± 2.1	698 ± 7.2	15.8 ± 0.9	52.7 ± 0.2	25.4	616 ± 3.5
S499F	43.2 ± 1.5	701 ± 6.8	16.2 ± 1.1	51.8 ± 0.1	21.5	618 ± 2.7
V280L/S499F	42.8 ± 1.7	708 ± 6.5	16.5 ± 0.9	54.2 ± 0.3	100.0	623 ± 1.9

### Structure analysis and molecular dynamic simulation of mutant enzymes for improving thermostability

**FIGURE 3:**
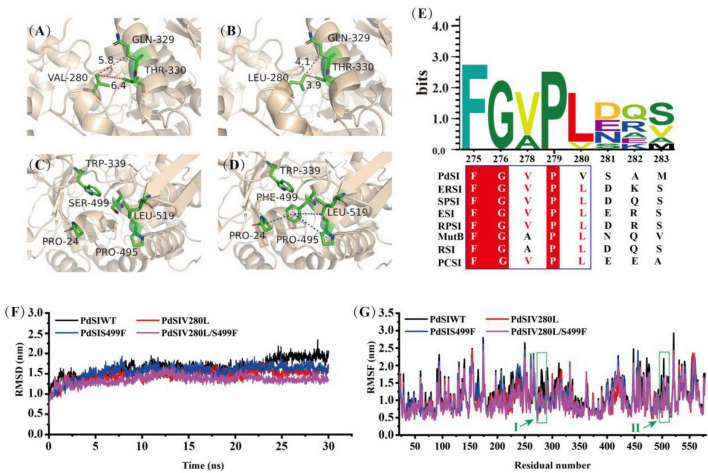
Structure analysis and Molecular dynamics simulations. **(A)** V280 was located in α-helix, forming two new VDWs with Q329 and W330 in mutant L280. **(B)** Reddish dashed lines refer to VDWs. **(D)** A new hydrophobic network including F499 and other residues (P24, W339, P495, and L519) was formed in S499 **(C)**, the hydrophobic interactions are shown as a blue dashed line, π-cation stacked interaction between Trp339 and Phe499 is shown as magentas line. **(E)** Multiple sequence alignment of SIases from different species. ERSI, *Erwinia rhapontici* NCPPB 1578; *Pantoea dispersa* UQ68J, PdSI; *Serratia plymuthica* PAMC26656, SPSI; ESI, *Enterobacter* sp. FMB-1; RPSI, *Raoultella planticola* UQ14S; MutB, *Pseudomonas mesoacidophila* MX-45; RSI, *Rhizobium* sp. MX-45 (M1E1F7); PCSI, *Pectobacterium carotovorum*. **(F)** The RMSD values of all backbone atoms for PdSIWT and mutants. **(G)** The RMSF values of each amino acid residue for PdSIWT and mutants. Letters I, II represent the amino acid position at site 280 and 499, respectively.

To analyze the conformational change of the mutations caused by each substituted residue, the 3D structure of PdSIWT and mutants were modeled with the Swiss-Model protein automated modeling program. The tight packing of protein interiors plays a vital role in protein stability for the burial of both polar and non-polar groups, and one -CH_2_- group buried on folding contributes 1.1 ± 0.5 kcal/mol of energy to protein stability (Nick Pace et al., 2014). As shown in Figure 3B, a single -CH_2_- group was added to the side chain after mutating the amino acid Val to Leu at position 280. This seems to reveal that the introduction of alanine’s bulky non-polar side chain may be responsible for improving the stability. In addition, an inspection of the structure model of the V280L showed that L280 was located in the α-helix (Figure 3A), and V280L substitution also generated two Vander Waals forces (VDW) bonds with Q329 and T330 of the other α-helix. Therefore, the two newly introduced VDW may also stabilize the local stability, thereby facilitating the geometry more stable. The thermostability of *Bacillus thermoleovorans* pullulanase was also successfully improved via the same strategy (Bi et al., 2020). Interestingly, all the amino acids at 280 sites from other sources were L except for V from the *P. dispersa* UQ68J through multiple sequence alignment (Figure 3E). Therefore, the increased thermostability of V280L mutant may also be related to the evolutionary conservatism of the enzyme (Pinney et al., 2021).

Previous studies have pointed out that molecular interactions, including hydrogen bonds, disulfide bonds, VDW, aromatic–aromatic interaction, and hydrophobic interaction, are the major structural factors that affect protein thermostability. In all of these factors, the contribution of hydrophobic interaction to protein stability accounts for about 60% (Gromiha et al., 2013). As shown in Figure 3D, a new hydrophobic network formed for substituted residue from hydrophilic S (Figure 3C) to strong hydrophobic F at site 499, which contains four residues (P24, W339, P495, and L519) within 5 angstroms. Therefore, residue 499 greatly changed the hydrophobic stacking around the mutation, enhancing the hydrophobic interaction effect. In addition, a cation–π interaction between F499 and W339 was found after mutation, which may further improve the thermostability of PdSIS499F. In summary, the improved thermostability of mutant PdSIS499F may result from the hydrophobic interactions and cation–π interaction. Moreover, no new molecular interactions were introduced into the double mutant PdSIV280L/S499F. Maybe the synergistic effect of these two single point mutations further promoted the improvement of stability of the double mutant.

In order to further clarify the overall structural rigidity of the enzyme and the fluctuation changes of each amino acid residue, we conducted MD of PdSIWT and three mutants (PdSIV280L, PdSIS499F, and PdSIV280L/S499F) at 318K for 30 ns in this study. Root mean square deviation (RMSD) and root mean square fluctuation (RMSF) represents the degree of molecular structure change and freedom of movement of individual atoms in a molecule, respectively. As shown in Figure 3F, the RMSD of all systems no longer fluctuates drastically after 13 ns, and then RMSD varied around 1.5 nm. After equilibration at 318 K, the average values of PdSIWT was 1.659 nm, whereas the average values of three mutants (PdSIV280L, PdSIS499F, and PdSIV280L/S499F) declined to 1.457, 1.520, and 1.353 nm, respectively. Since the thermostability of protein is not positively correlated with its RMSD value, the lower RMSD value of mutants indicated the mutated structure was relatively stable than that of the PdSIWT.

Similarly, RMSF could also reflect the local flexibility of the protein. One region has a higher RMSF value, indicates that the conformation of this region was more unstable. As shown in Figure 3G, some regions around residue V280, S499 showed significant fluctuations in RMSF values of PdSIWT at 318 K. Generally, these amino acids were thought to be thermo-unstable. On the contrary, RMSF of three mutants (PdSIV280L, PdSIS499F, and PdSIV280L/S499F) showed mild fluctuations in the same areas of PdSIWT mentioned above. In conclusion, mutations in these sites (V280 and S499) contribute significantly to improving the stability of PdSIWT.

### Development of a one-step simplified immobilization method for *C. glutamicum* cells

**FIGURE 4:**
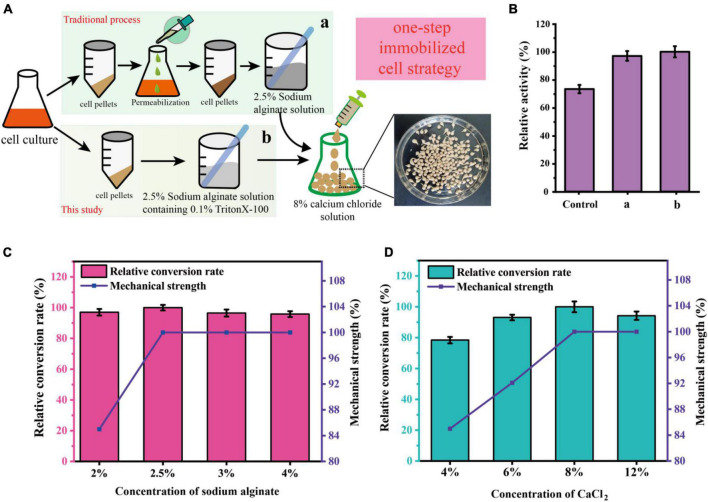
Schematic representation of the immobilization of *C. glutamicum* cells using a one-step simplified method. **(A)** Procedures for immobilization of *C. glutamicum* cells. **(B)** Comparison of different immobilization methods. [Control: without cell permeabilization, (a) cell pre-permeabilization with 0.1% triton-X100 before immobilization, (b) 2.5% Sodium alginate solution containing 0.1% triton-X100 was used for cell immobilization]. **(C)** Effect of different sodium alginate concentrations on sucrose conversion and mechanical strength of immobilized pellets. **(D)** Effect of different CaCl_2_ concentrations on sucrose conversion and mechanical strength of immobilized pellets.

To develop an economically feasible immobilization method for *C. glutamicum* cells, several different immobilization methods were investigated in this work. As shown in Figure 4A, the traditional immobilization of *C. glutamicum* cells usually requires centrifugation followed by permeabilization, which is cumbersome in industrial production (a). Therefore, reducing the operation steps of immobilization is more in line with the requirements of industrial production (b). After research and comparison (Figure 4B), it was found that shortening the process of immobilization did not affect the catalytic effect, and even the catalytic efficiency was better than that of traditional immobilization. The relative activity of the control (without permeabilization) and a (pre-permeabilization) reached 73.3 and 97.2%, respectively, compared to the b (without pre-permeabilization) in this study.

To further optimize the preparation of immobilized *C. glutamicum* cells, we further optimized the concentration of sodium alginate and CaCl_2_. As shown in Figure 4C, an increase of initial sodium alginate concentrations from 2.0 to 2.5% increased the conversion rate and mechanical strength (435 g/cm^2^). However, the mechanical strength (435 g/cm^2^) stopped increasing and the conversion rate began to decline, when the initial sodium alginate concentrations exceeded 2.5%. Also, the concentration of CaCl_2_ plays a huge role. As shown in Figure 4D, the mechanical strength is greatly affected by the concentration of CaCl_2_, increasing concentrations of CaCl_2_ from 4.0 to 8.0% markedly increased the conversion rate and mechanical strength. At 8.0%, the conversion rate and mechanical strength (485 g/cm^2^) are optimal. Thus, the subsequent preparation of immobilized *C. glutamicum* cells was carried out under this condition.

### Optimization of reaction conditions for the immobilized RCSI1 and RCSI2 biotransformation

**FIGURE 5:**
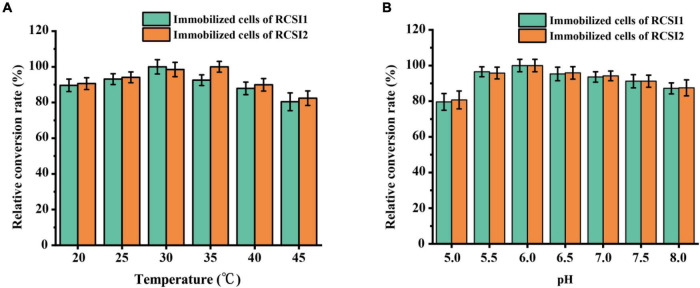
Optimization of biotransformation conditions for isomaltulose production using immobilized *C. glutamicum* cells. **(A)** Effect of temperature. **(B)** Effect of pH.

To optimize the biocatalytic conditions for isomaltulose production, the influence of temperature, pH as variables were explored using immobilized RCSI1 (recombinant cells expressing wild-type SI) and RCSI2. As shown in Figure 5A, the optimum pH of immobilized RCSI1 and RCSI2 was pH 6.0, no noticeable changes were detected in the optimum pH conditions. However, shift in the optimal temperature was observed between immobilized RCSI1 and RCSI2, the relative activity of RCSI1 was highest at 30°C, while the maximum catalytic activity of RCSI2 was at 35°C (Figure 5B). At the same time, we also observed that immobilized RCSI2 exhibited a broad catalytic capacity in the range of 25–35°C. Therefore, the phenomenon may be attributed to the differences in the micro-environment after encapsulating the engineered thermostable PdSIV280L/S499F into immobilized recombinant *C. glutamicum* cells.

### Batch culture of RCSI2 and sustainable synthesis of isomaltulose in a 5 L fermenter

**FIGURE 6:**
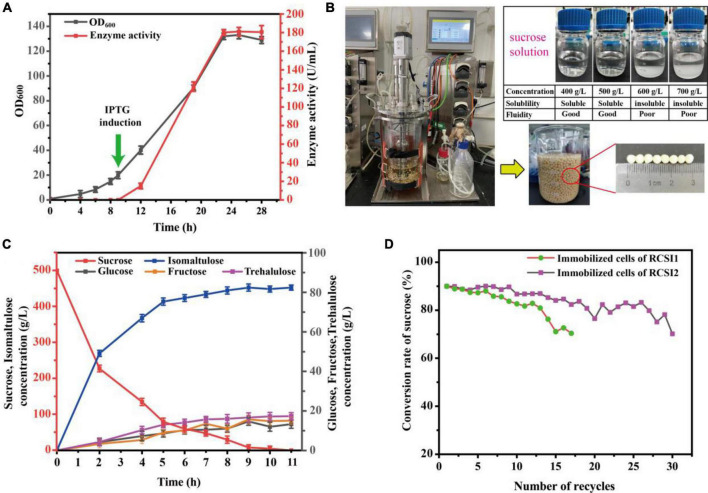
Batch culture and biotransformation were performed in a 5 L fermenter. **(A)** Batch culture of strain RCSI2 in a 5 L bioreactor. **(B)** Efficient production of isomaltulose using immobilized *C. glutamicum* cells in 5 L fermenter. **(C)** Time course of the isomaltulose production from the sucrose in 5 L fermenter. **(D)** Sustainable synthesis of isomaltulose using immobilized *C. glutamicum* cells.

Batch culture of strain RCSI2 was tested in a 5 L fermentor. The DO was controlled at 30% during the whole process by coupling the rotation. As shown in Figure 6A, the cell density of OD_600_ increased to 19.3 in initially 9 h, and then IPTG was supplemented to the bioreactor at a final concentration of 0.5 mM to induce the expression of SI at this time. Subsequently, the biomass increases rapidly, OD_600_ increased to 132.2 and the total enzyme activity reaches 180.2 U/mL in 23 h. After 28 h, the cell density of OD_600_ started to drop to 129.5, and the enzyme activity (180.2 U/mL) no longer increased at this time. Finally, the cells were centrifuged for subsequent immobilization.

Based on the above results, one-batch biocatalysis process was applied to transform sucrose to isomaltulose in a 5 L fermenter using immobilized RCSI2 cells. However, limited by the solubility of the substrate (Figure 6B), 500.0 g/L sucrose was selected for isomaltulose production in this study. As shown in Figure 6C, the reaction time-course curves, consisting of isomaltulose production and by-product glucose and fructose accumulation, are illustrated. The concentration of isomaltulose increased rapidly for the first 6 h and gradually reached a plateau after 11 h. To be specific, the maximum yield of isomaltulose reached 453.0 g/L in 11 h with a conversion rate of 90.6% (w/w) and a productivity of 41.2 g/L/h. At the same time, 13.3g/L glucose, 15.1 g/L fructose and 17.3 g/L trehalulose were produced as byproduct during the reaction.

To increase isomaltulose productivity and save the cost of culturing bacteria, the sustainable catalytic reaction of immobilized cells was evaluated. As depicted in Figure 6D, the immobilized RCSI2 exhibited robust and excellent operational stability with a total reaction batches up to 30 and maintained more than 83.2% of the initial isomaltulose productivity even after 26 batches of repeated utilization. However, the conversion rate of RCSI1 decreased to 71.1% after 15 batches. Therefore, we successfully obtained a recombinant GRAS strain with the highest operational stability. The productivity was also the highest reported among the food-grade strains (Table 3), still 1.1 times higher than the highest previous study on recombinant *B. subtilis* strains.

**TABLE 3:** Comparison of isomaltulose yield in different food grade strains.

Strains	Concentration of sucrose (g/L)	Yield of isomaltulose (g/L)	Productivity (g/l/h)	Number of rescue	Conversion rate	References
**Immobilized Enzyme**						
*Y Yarrowia lipolytica* XY	–	–	–	13	∼80%	Zhang et al., 2019
**Whole-cell**						
*Yarrowia lipolytica* S47	600	572.1	23.83	1	95%	Zhang et al., 2018
*B. subtilis*	230.1	221.6	36.9	12	∼80%	Wu et al., 2017
*Yarrowia lipolytica* CGMCC7326	500	460	32.8	12	∼80%	Li et al., 2017
*S. cerevisiae*	50	<4	0.09	1	<0.08	Lee et al., 2011
*L. lactis*	50	36	0.75	1	72%	Park et al., 2010
*Corynebacterium glutamicum*	500	453	41.2	26	83.2%	This study

## Conclusion

The outstanding thermostability of SI has always been pursued in successful industrial manufacturing bioprocess for isomaltulose, as even a slight enhancement can improve long-term activity under reaction conditions and increase the ability to remain high activity in the biotransformation. In this work, the thermal stability of SI from *P. dispersa* UQ 68J toward sucrose isomerization was greatly improved via rational engineering utilizing computer-aided design and conservation analysis and functional region assessment. We gained a robust variant PdSIV280L/S499F, which displayed a 3.6°C increase in apparent melting temperature and about ninefold longer half-life at 45°C compared to PdSIWT. More importantly, we characterized the reason underlying increased thermostability systemically. These results showed that the comprehensive strategy is a versatile and efficient method to improve the thermostability of enzymes without extensive experiment. In addition to the properties of the enzyme itself, the catalytic method is equally important. Immobilized cell transformation is the most promising catalytic method for industrial production of isomaltulose, which cannot only improve long-term activity under optimum conditions, but also facilitates sustainable production. However, immobilization of Gram-positive bacterial cells is still complicated and tedious. Therefore, we also developed a one-step simplified cell immobilization, the immobilized RCSI2 catalyst exhibits robustness and continuous operational stability in isomaltulose production, the conversion rate remained at 83.2% even after 26 continuous rounds of biocatalysis. In conclusion, protein engineering coupled with a one-step immobilized cell strategy provides an effective method to enhance the high yield of isomaltulose in this study.

## Data availability statement

The datasets presented in this study can be found in online repositories. The names of the repository/repositories and accession number(s) can be found in the article/Supplementary material.

## Author contributions

MH designed and performed the experiments and wrote manuscript. ZW and FL analyzed bioinformatics data. MS, MX, TY, and RZ performed investigation and resources. XZ edited and revised the manuscript. ZR provided project administration and funding acquisition. All authors contributed to the article and approved the submitted version.

## Footnotes

1
http://www.yasara.org
