# Supplementary material for: Sustainable isomaltulose production in Corynebacterium glutamicum by engineering the thermostability of sucrose isomerase coupled with one-step simplified cell immobilization
Source: Front Microbiol. 2022 Aug 10;13:979079. doi: 10.3389/fmicb.2022.979079 (PMC9399683; doi:10.3389/fmicb.2022.979079)
Supplement: Supplementary file 1 [file Data_Sheet_1.docx]

**Supporting Information**

**Sustainable isomaltulose production in *Corynebacterium glutamicum* by engineering the thermostability of sucrose isomerase coupled with one-step simplified cell immobilization**

Mengkai Hu^1^, Fei Liu^1^, Zhi Wang^1^, Minglong Shao^1^, Meijuan Xu^1^, Taowei Yang^1^, Rongzhen Zhang^1^, Xian Zhang^1*^, and Zhiming Rao^1*^

1Key Laboratory of Industrial Biotechnology of the Ministry of Education, School of Biotechnology, Jiangnan University, Wuxi, Jiangsu 214122, China

Keywords: isomaltulose, thermostability, sucrose isomerase, one-step simplified immobilization, repeated batches

*Corresponding authors:

Prof. Xian Zhang, E-mail: zx@jiangnan.edu.cn, Tel：13771401977,

Prof. Zhiming Rao, E-mail: raozhm@jiangnan.edu.cn, Tel：13921135816

**
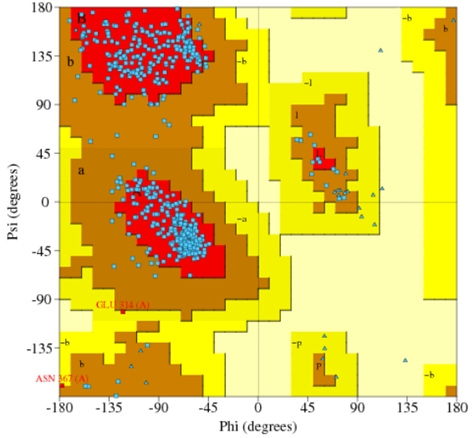
**

**Figure S1.** Ramachandran plot of *Pantoea dispersa* UQ68J sucrose isomerase model. The preferred regions, allowed and disallowed regions were delineated with red, yellow and white, respectively. The results showed that 99.6% and 0.4% of residues were located in allowed regions and generously allowed regions, respectively..


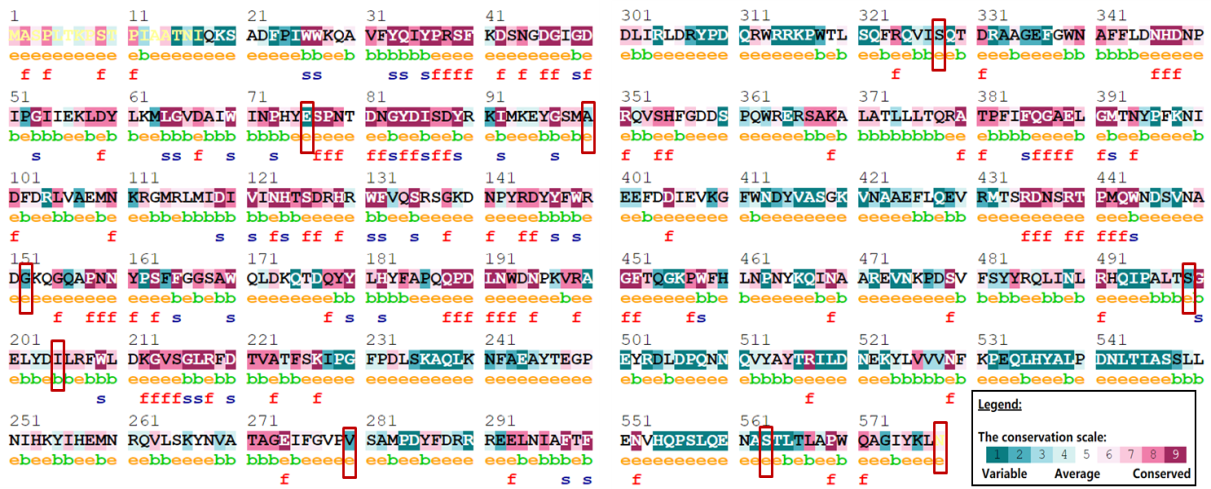


**Figure S2.** The evolutionary conservation analyses of *Pantoea dispersa* UQ68J sucrose isomerase using the ConSurf server. The letter e represents an exposed residue according to the neural-network algorithm, letter b represents a buried residue according to the neural-network algorithm, letter f represents a predicted functional residue (highly conserved and exposed), letter s represents a predicted structural residue (highly conserved and buried), letter x represents Insufficient data-the calculation for this site was performed on less than 10% of the sequences. The potential mutation sites are shown in red box.

**Table S1.** Strains and plasmids used in this study

| Plasmids/Strains | Genotype or sequence | Source |
| --- | --- | --- |
| Plasmids |  |  |
| pXMJ19 | Expression vector, T_tac_ promoter, chl^r^ | Lab collection |
| pXMJ19-*Pdsi* | pXMJ19 derivative; T_tac_ promoter, the synthetic *Pantoea dispersa* UQ68J *si* gene; chl^r^ | This study |
| Strains |  |  |
| *E. coli* JM109 | Applied for cloning gene | Lab collection |
| *C. glutamicum* ATCC13032 | Applied for expressing gene | Lab collection |

**Table S2.** The main primers for site-directed mutagenesis used in this study

| primers | oligonucleotide（5′-3′） |
| --- | --- |
| E76R-F | CCGCACTAC***CGC***AGTCCGAATACCGACAACGGC |
| E76R-R | TTCGGACT***GCG***GTAGTGCGGGTTGATCCAGAT |
| A100E-F | GAGTATGGCAGCATG***GAA***GACTTTGACCGTTTA |
| A100E-R | TAAACGGTCAAAGTC***TTC***CATGCTGCCATACTC |
| G152P-F | TTTTGGCGCGAC***CCG***AAACAAGGTCAAGCTCCGAAT |
| G152P-R | TTGACCTTGTTT***CGG***GTCGCGCCAAAAGTAGTAGTC |
| I205M-F | GCCGAGCTGTACGAC***ATG***TTACGCTTCTGGCTG |
| I205M-R | CAGCCAGAAGCGTAA***CAT***GTCGTACAGCTCGGC |
| V280L-F | ATCTTTGGTGTTCCG***CTG***AGCGCCATGCCCGAT |
| V280L-R | ATCGGGCATGGCGCT***CAG***CGGAACACCAAAGAT |
| S328F-F | TTCCGCCAAGTTATC***TTC***CAAACCGATCGTGC |
| S328F-R | GCACGATCGGTTTGG***AAG***ATAACTTGGCGGAA |
| S499F-F | ATCCCGGCACTGACC***TTC***GGCGAATACCGCGA |
| S499F-R | ATCGCGGTATTCGCC***GAA***GGTCAGTGCCGGGAT |
| S563L-F | CTGCAAGAAAATGCC***TTA***ACCTTAACTTTAGCCCC |
| S563L-R | GCTAAAGTTAAGGT***TAA***GGCATTTTCTTGCAGACTCGG |
| S563R-F | CTGCAAGAAAATGCC***CGC***ACCTTAACTTTAGCC |
| S563R-R | GGCTAAAGTTAAGGT***GCG***GGCATTTTCTTGCAG |
| N578M-F | GGCATTTACAAATTA***ATG***CACCACCACCACCA |
| N578M-R | TGGTGGTGGTGGTG***CAT***TAATTTGTAAATGCC |

Notes: Mutation sites are shown in bold and Italic
